# Evidence based policy making and the ‘art’ of commissioning – how English healthcare commissioners access and use information and academic research in ‘real life’ decision-making: an empirical qualitative study

**DOI:** 10.1186/s12913-015-1091-x

**Published:** 2015-09-29

**Authors:** Lesley Wye, Emer Brangan, Ailsa Cameron, John Gabbay, Jonathan H. Klein, Catherine Pope

**Affiliations:** Research Fellow, School of Social and Community Medicine, University of Bristol, Canynge Hall, 39 Whatley Road, BS8 2PS Bristol, UK; School of Social and Community Medicine, University of Bristol, Bristol, UK; School of Policy Studies, University of Bristol, Bristol, UK; Wessex Institute for Health Research and Development, University of Southampton, Southampton, UK; Southampton Business School, University of Southampton, Southampton, UK; Faculty of Health Sciences, Southampton, UK

**Keywords:** Evidence-based policy-making, Evidence, Commissioner, Research, Information seeking

## Abstract

**Background:**

Policymakers such as English healthcare commissioners are encouraged to adopt ‘evidence-based policy-making’, with ‘evidence’ defined by researchers as academic research. To learn how academic research can influence policy, researchers need to know more about commissioning, commissioners’ information seeking behaviour and the role of research in their decisions.

**Methods:**

In case studies of four commissioning organisations, we interviewed 52 people including clinical and managerial commissioners, observed 14 commissioning meetings and collected documentation e.g. meeting minutes and reports. Using constant comparison, data were coded, summarised and analysed to facilitate cross case comparison.

**Results:**

The ‘art of commissioning’ entails juggling competing agendas, priorities, power relationships, demands and personal inclinations to build a persuasive, compelling case. Policymakers sought information to identify options, navigate ways through, justify decisions and convince others to approve and/or follow the suggested course. ‘Evidence-based policy-making’ usually meant pragmatic selection of ‘evidence’ such as best practice guidance, clinicians’ and users’ views of services and innovations from elsewhere. Inconclusive or negative research was unhelpful in developing policymaking plans and did not inform disinvestment decisions. Information was exchanged through conversations and stories, which were fast, flexible and suited the rapidly changing world of policymaking. Local data often trumped national or research-based evidence. Local evaluations were more useful than academic research.

**Discussion:**

Commissioners are highly pragmatic and will only use information that helps them create a compelling case for action.Therefore, researchers need to start producing more useful information.

**Conclusions:**

To influence policymakers’ decisions, researchers need to 1) learn more about local policymakers’ priorities 2) develop relationships of mutual benefit 3) use verbal instead of writtencommunication 4) work with intermediaries such as public health consultants and 5) co-produce local evaluations.

**Electronic supplementary material:**

The online version of this article (doi:10.1186/s12913-015-1091-x) contains supplementary material, which is available to authorized users.

## Background

Since the mid-1990s, policymakers have been encouraged to increase their use of evidence [1]. For researchers, ‘evidence-based policy-making’ or ‘evidence-based decision-making’ usually means drawing on academic research such as systematic reviews, economic evaluations, randomised controlled trials and (to a lesser extent) qualitative studies [2, 3]. This research may be clinical or managerial. But some prominent researchers have called for caution. As early as 2000, Klein warned that the emphasis on evidence-based policy-making exaggerated the claim of what academic research could deliver and “grossly misunderstood” the policy process [4].

Those charged with delivering ‘evidence-based policy-making’ within the English National Health Service include local healthcare commissioners that sit within organisations called Clinical Commissioning Groups. Currently there are over 200 local Clinical Commissioning Groups across England that are responsible for about £110 billion annually. Clinical Commissioning Groups plan and allocate funding for healthcare providers from hospital and community services. A major objective of the recent reorganisation of the National Health Service (Health and Social Care Act 2012) was to increase the level of clinical input into commissioning [5]. Within Clinical Commissioning Groups, local general practitioners (GPs) often take on lead clinical roles within their areas of special interest (e.g. long-term conditions, unplanned hospital admissions), working closely with commissioning managers without clinical backgrounds. To inform their decisions, Clinical Commissioning Groups need support from many other organisations including public health, which now are located within the local council (town hall), and Commissioning Support Units, which provide a variety of functions including contracting, business intelligence (i.e. hospital and community service data analysis) and project management. Other local and national organisations within this landscape include social care, which sits within local councils, commercial and not-for-profit providers that offer software tools and consultancy, the National Institute for Clinical and Healthcare Excellence (NICE), which reviews research evidence and generates guidance about effective (and ineffective) treatments and NHS Improving Quality, formerly the NHS Institute for Innovation and Improvement, that promotes quality improvement programmes and tools. Above Clinical Commissioning Groups sits NHS England, which is charged with commissioning specialist services for less common conditions and until recently, also commissioned general practices. NHS England, along with the Department of Health, also offer strategic direction for the entire National Health Service, issuing regular guidance and compulsory directives. Within this complex environment, healthcare commissioners have to locate the information needed to best inform their decisions.

Few studies of local healthcare commissioners have taken place, and the majority are interview based [6]. They have suggested that healthcare commissioning is “messy and fragmented” and largely accomplished in meetings [7]. Those meetings can take many forms including informal chats between internal colleagues, more formal contract and quality monitoring between commissioners and healthcare providers, exploratory discussions between commissioners, providers and sometimes public health and the commissioning support unit to develop or modify services, regular monthly meetings of senior commissioners on the ‘leadership team’ and public meetings of the ‘governing body’ which include senior directors and the governing boards. Progress is made incrementally in “bite-sized pieces of work” requiring substantial effort [8] and that considerable ingenuity and local knowledge is required to create something “meaningful” and “intelligent” from national top-down policies [9], which can be subverted to meet local agendas [10]. Mid-level managerial commissioners have a key role, in that they are responsible for transferring, summarising and interpreting information to their peers and to senior decision makers [11]. However, because there is limited research into healthcare commissioning, those outside of commissioning circles do not know much about how commissioners draw on academic research and other information in actuality.

Learning more about how and where commissioners access information is important to increase understanding about the contribution that researchers and research evidence can make to policy-making. Using observational, interview and documentary data, this paper offers ‘real life’ findings from one of the first studies of clinical commissioning under these new arrangements in the English NHS. The aims of the study are to elucidate the reasons that prompted commissioners to seek information, to clarify which sources and types of knowledge commissioners commonly consulted and to describe the use of research evidence in decision-making. Throughout this paper, we use the terms ‘knowledge’, ‘information’ and ‘evidence’ interchangeably, as commissioners do, and we use the term ‘research’ to distinguish academic research. Although knowledge acquisition (the ways that individuals obtain information) and knowledge transformation (the ways that individuals modify information to make it usable) are different concepts, in practice we found the two happened concurrently, but for the sake of clarity we focus here on knowledge acquisition; findings on knowledge transformation are published in the full study report [12] and another paper [13].

## Methods

### Study design

The data presented here come from a larger study of knowledge exchange between commissioners and external providers such as commercial companies. We used a mixed qualitative methods case study approach [14]. The study received ethical permission from South West Ethics Committee 2 (10/H0206/52).

### Case site selection

We could only selected those commissioning organisations that had contracts with the commercial providers that participated in the larger study. In total, these commercial providers had less than a dozen contracts with over 12 different commissioning organisations that were geographically located across the country. We selected the commissioning organisations on the basis of geography (north, south and London), size (small/large), population (urban/rural) and length of time with contracted provider (early, mid and post-contract). Using pseudonyms, the four commissioning organisations selected were:

- Carnford - struggling financially and highly collaborative with its healthcare providers
- Deanshire – relatively confident as a commissioning organisation, focused on governance, carrying out some innovative projects
- Norchester - financially challenged, piloting new ways of commissioning contracts with an emphasis on (ideally academic research) evidence-based policy making
- Penborough – creating an integrated network of health and social care provision with an emphasis on public involvement and innovation

### Data collection

We collected interview, observation and documentary data from February 2011 to May 2013. Data were collected by LW and EB, who are experienced qualitative researchers.

The contracts between the commissioning organisation and external providers involved in the larger study of knowledge exchange were the focus of our sampling strategy. Through snowball sampling, we identified candidates for interview from within commissioning organisations, such as the lead NHS contact for the contract. As our enquiry broadened, we identified those with particular roles who could further illuminate the knowledge acquisition behaviours of commissioners (e.g. chairs of the commissioning organisation, directors, public health consultants).

Candidates were sent information before interviews and written or recorded consent was obtained at interview. The topic guide, which was revised as new questions emerged, covered the types of knowledge wanted by commissioners, information sources and how information was accessed and influenced decisions. Face to face and telephone interviews lasted 20–60 min; they were recorded and transcribed by an external transcriber. In total, we interviewed 52 people (Table 1).

**Table 1:** Interview participants

Professional role	Number of participants
Managerial commissioner	17
Clinical commissioner	15
Information Analyst	9
Other NHS	6
Public health consultants	4
Local authority commissioner	1
Total	52

We conducted 14 observations of commissioning meetings including director-level boards (*n* = 7), clinical operations committees (*n* = 3), unscheduled-care boards (*n* = 3) and a project board (*n* = 1). Permission was obtained verbally before attending meetings and re-confirmed at the start of each meeting. Observation notes included details of participants, room layout, verbal exchanges and researcher reflections. Notes were usually typed up within a week. All interview and observation participants were given pseudonyms.

Documentary evidence included board papers, meeting minutes, reports, websites and e-mails, which were collected and fed into the case summaries (see data analysis). These supplemented, confirmed and challenged emerging findings from interview and observation data.

### Data analysis

Constant comparison methods guided the analysis, whereby data were compared across categories, which were continually refined and fed back into data collection (and analysis) cycles [15]. The study team met regularly to identify emerging themes, reflect on the research questions and suggest new questions for the topic guide. In May 2013, fieldwork came to a close and team members (EB, LW and AC) developed a coding framework based on the research questions for the larger study, which was applied by both EB and LW to guide the initial analysis. Using NVIVO software, we systematically coded cases and developed 20–50 page summaries for each case structured around several domains including knowledge acquisition. Every member of the research team read these summaries independently and conducted cross case analyses to identify key themes and discrepant data. The team then met to agree key themes and develop interpretations.

## Results

Our results are structured along the following themes identified in the analysis:

- Reasons that commissioners seek information
- Sources and types of information sought
- Ways that information was exchanged
- Use of academic research and local evaluations

### Why do commissioners want information?

Commissioners purposefully sought out information either because they were told to take a particular course of action (e.g. national directives from the Department of Health or NHS England) or because they wanted to find out how best to proceed and no predetermined course existed. Both occurred frequently across the sites. In both scenarios, commissioners used information to try to build a compelling, cohesive and persuasive case to a) navigate a way through the system b) justify decisions and c) convince others to approve and/or follow the suggested course. Decision-making occurred through repeated cycles of finding information, persuading others, justifying decisions, finding more/different information, persuading others etc., as proposals moved through the commissioning decision-making process.

Regardless of the motivation for information seeking, because commissioning decisions were publically accountable as commissioning organisations are statuatory bodies, they had to “stand up to extremely close, possibly legal scrutiny and have to be owned by the organisation” (Angus, GP commissioner). Decisions had to be resilient to challenges from many possible directions. These included challenges from:

- clinicians and healthcare provider organisations (e.g. hospitals, community service providers such as district nursing) needing to make the necessary changes
- service users and the public, for example to smooth the introduction of potentially unpalatable changes to services
- the press to avoid negative media attention
- ‘evidence purveyors’ such as public health that lobbied commissioning organisations

Substantial pressure also came from those with a policy or performance management role, especially senior policymakers such as the Department of Health and/or NHS England area teams. Local circumstances dictated the extent to which such top-down attention was welcomed. For example, several board documents from one commissioning organisation noted that local activities had substantial “interest from Number 10” [i.e. the Prime Minister’s office], which spurred momentum. But sometimes this was more problematic, as with one commissioning organisation’s response to the national mandate to tender a specific list of services to open competition, which proved troublesome as this organisation was happy with their services in these areas.

*Again it was an example of centralisation of things coming down from above. They said that we had to put out two or three services to any qualified provider [open competition]. So [commissioning organisation X] decided what we should do. And one [option] was ultrasound, non-obstetric ultrasound, which we have an absolutely excellent service provided by the hospital, even routine ones are done within a week, brilliant service. So why do we do it? So there’s all this process and people – they ended up with a list of seven providers. But, you know, it was a complete waste of time and money. (David, CCG Vice-Chair, Penborough)*

This organisation routinely resisted national directives from the Department of Health and NHS England and consequently experienced pressure to comply.

*GP commissioner: I just see how the world works and the pressure that organisations are put under if they don’t conform. You know, these words like, “You are at risk. Your organisation is at risk. You are at personal risk for this.” And that’s not a nice thing to be on your shoulders….*

*Interviewer: Yeah, so you’re identified as risky?*

*GP commissioner: Yeah and then you get lots of phone calls, and the chief exec gets phone calls, and he has to speak to you going, “Oh I’m getting a lot of flak about this. Can you not just smile sweetly and say you’ll engage?” (Vidur, GP commissioner, Penborough)*

In addition to managing these external forces, commissioners also had to convince their internal colleagues. For example at one board meeting of senior commissioners, a junior commissioning manager putting forward a business case for a lymphoedema service costing £92,000 annually. This was up against several other competing proposals suggested by colleagues from the same organisation, each trying to persuade the board that their particular business case should be funded.

**Fig. 1:**
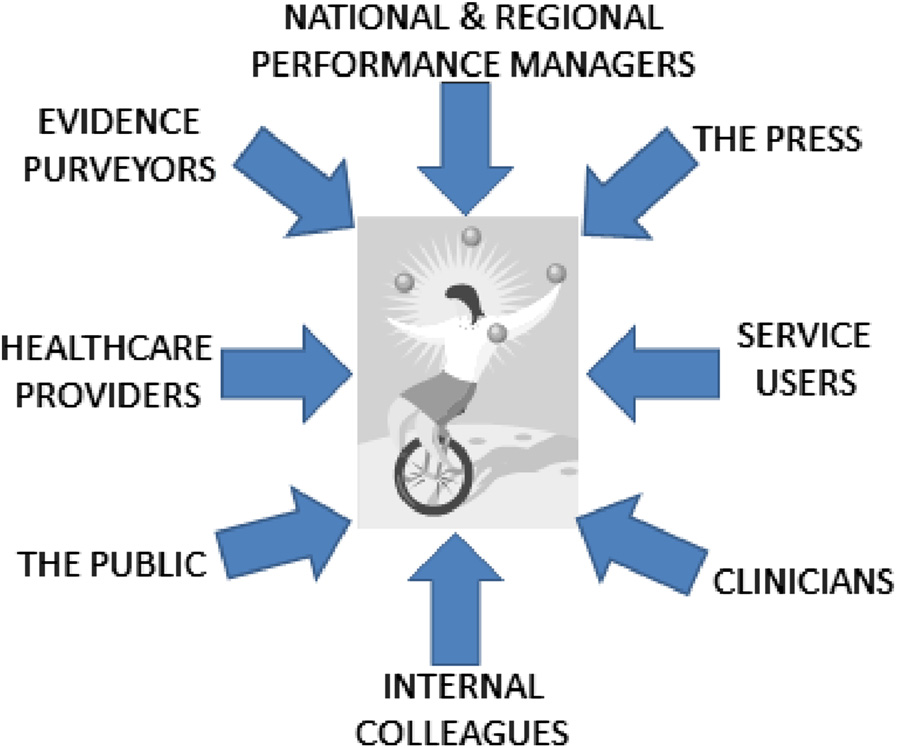
Pressures on commissioners

In summary, commissioners had to influence and collaborate with external and internal interested parties to build a compelling case for taking a particular course of action. Not all challenges came into play, and there was also variability in the strength of each as a proposal traversed the different stages in the decision-making process. But invariably, at the centre of this web of pressurising forces, the commissioner juggled competing agendas, priorities, power relationships, demands and their own inclinations to make the best decision circumstances allowed. Just as there is an ‘art of medicine’, this was the ‘art of commissioning’ (see Fig. 1).

To a large extent, commissioning was a matter of pragmatically pulling together the appropriate knowledge and information that would satisfy competing agendas and help manoeuvre the proposal. Unsurprisingly, as that knowledge was juggled and steered, it was re-considered, re-framed and re-shaped, usually becoming quite different as a result. For example, an evaluation of a rehabilitation project for those with long term conditions was carried out by public health showed a small reduction in hospital admissions for service users after the intervention, but offered no comparable results for the comparison group (see Additional file 1 for full account). The briefing paper authored by public health reported that the cost of social care packages went up for both the service user and control groups, although it was significantly less for service users. These findings were presented to the board, but the minutes recorded quite different results.

*The principal outcomes of the study indicated: a significant difference between the patient groups in the number of hospital re-admissions and a significant reduction in social care costs. (Meeting minutes, Deanshire)*

**Fig. 2:**
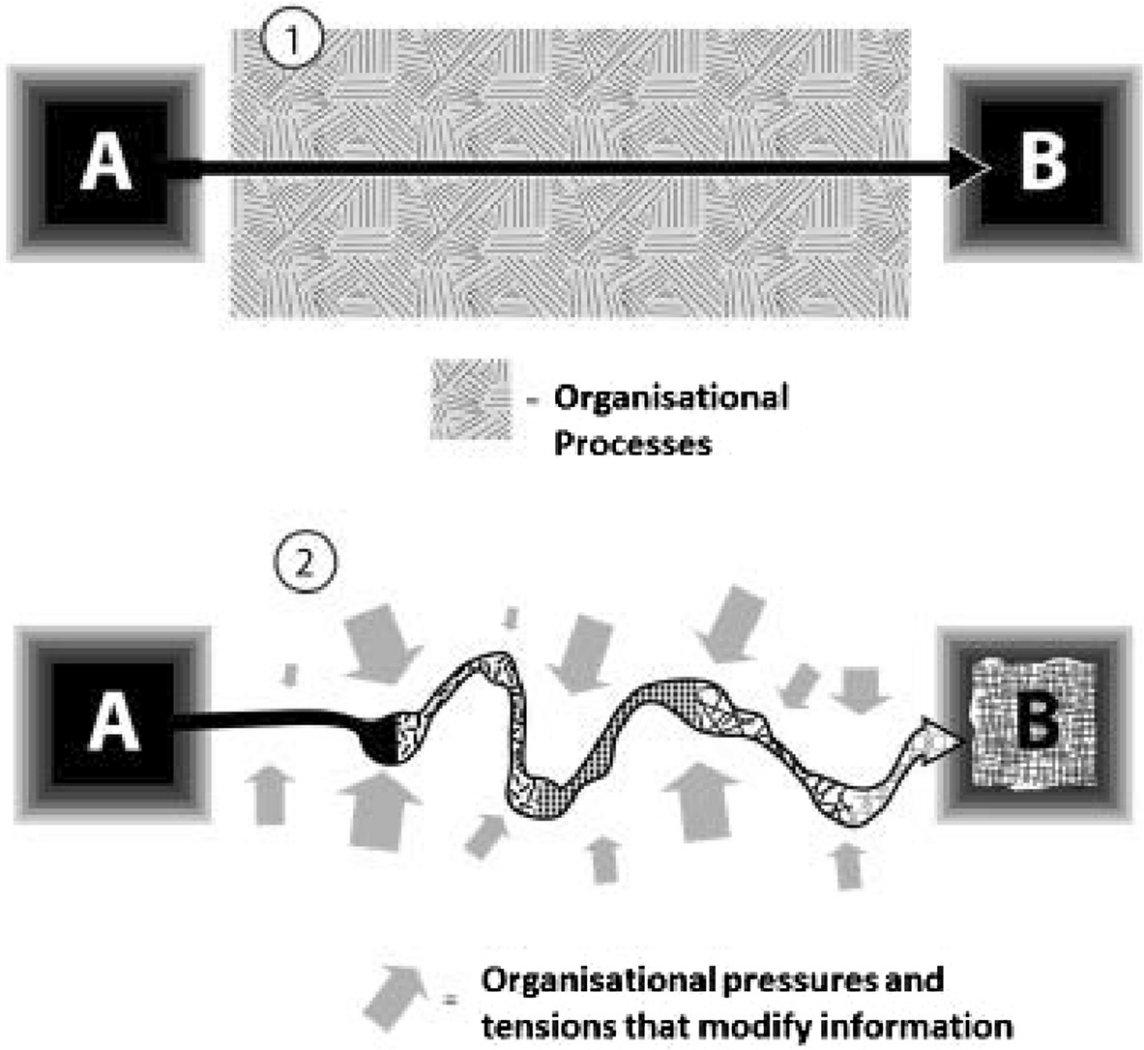
Information as it moves through the system: the naïve view (1) and the view suggested by our data (2)

Partly on the basis of this over-statement of the original evidence about the impact of the intervention on hospital utilisation and costs, the project was re-commissioned at £250,000 annually when its business case was presented. Such modifications of evidence in the decision-making processes was not untypical. Figure 2 illustrates how information was regularly re-shaped, where A is the original information and B is the information as it moves through the system. It contrasts the naïve view that the original information glides through unaffected by organisational processes with a depiction of how organisational processes change the original information (Fig. 2).

### What information do commissioners access?

Commissioners sought knowledge and information from many sources to determine, clarify, substantiate and defend their case. For example, in re-designing new services for a patient pathway, commissioners might use:

- local clinicians’ knowledge of services
- analysts’ information about service referral rates
- service users’ experiences of their condition
- NICE clinical guidelines
- NHS Improving Quality guidance on best practice
- Public health data on trends
- *Health Service Journal* (a leading weekly professional journal) for examples of similar pathways devised by other commissioning organisations

For commissioners, the word ‘evidence’ often meant any source of information other than personal experience or anecdotes. When asked, they commonly named NICE guidelines, best practice guidance, hospital and primary care data and Department of Health documentation as sources of ‘evidence’. An overview of the sources and types of knowledge cited by commissioners and observed in commissioning meetings is detailed in the following tables, classified into ‘people’ and ‘organisations’. These are not exhaustive, as other sources and types of knowledge undoubtedly exist and the named sources offer other types of knowledge not directly referenced by participants. As this is not a quantitative survey [16], we make no claims to the representativeness of this classification nor attribute weightings to the particular factors but use this to illustrate the wide range of sources referred to (Tables 2 and 3).

**Table 2:** People-based sources of information and types of knowledge mentioned in interviews or observed

	Local clinicians	Commissioning managers	Analysts	Patients and the public	Freelance consultants
Local relationships	✓	✓	✓	✓	✓
Local history	✓	✓	✓	✓	✓
Experiential knowledge^a^	✓	✓	✓	✓	✓
How services operate	✓	?		✓	
‘Whole picture view’		✓			
Hospital, primary &/or community data	✓	?	✓		✓
Data interpretation & what data to trust	✓	?	✓		
Local benchmarking		?	✓		
Project based experience		✓	?		✓
Condition specific expertise	✓			✓	
Software tool operation			✓		✓
Contracting, procurement, finance & budgets		✓			✓
Academic research	?				

? = to some extent

^a^Type of experiential knowledge varied depending on source e.g. commissioning managers knew how to steer information through the system and patients & the public knew how to apply pressure through complaints and media campaigns

**Table 3:** Organisation-based sources and types of knowledge mentioned in interviews or observed

	Department of Health	NICE	NHS Improving Quality	Public health	CSU	‘Think tanks’ e.g. Kings Fund	Royal Colleges	Local healthcare providers	Non-local CCGs/ CSUs & healthcare providers	Commercial & not-for- profit providers
Best practice	✓		✓			✓	✓		✓	✓
Local relationships					✓					
Electronic newsletters			✓			✓				
Organisational development					✓	✓				✓
Commissioning guidance	✓					✓	?			
Clinical guidelines		✓					✓			
‘Horizon scanning’										✓
Academic research		✓		✓		✓				
Improvement tools			✓							✓
Project management					✓					✓
Service &/or population data				✓	✓			✓		
Commissioning experience				✓		✓			✓	✓
Products										✓
Patient pathways								✓	✓	
Advanced data = interrogation skills										✓

Amongst national sources, commissioners appeared to trust NICE, the King’s Fund and NHS Improving Quality (formerly NHS Institute for Innovation and Improvement), which were perceived as independent. Clinical commissioners were more sceptical of output from the Department of Health, sometimes because they felt that this ‘evidence’ did not stand up to scrutiny.

Local information often trumped national evidence, academic research or information from other localities. For example, ‘telehealth’ was a national ‘must do’ with limited clinical support in one commissioning organisation. An unscheduled-care board was presented with a review of the academic research behind ‘telehealth’ presented by a public health consultant. This suggested weak evidence of clinical and cost effectiveness. A month later, the board became more enthusiastic, when provided with before and after data from a local evaluation of just the first eight local patients.

*Cindy: we’re not entirely sure but it seems to have some evidence that something happening…It’s soft measures such as empowerment. Lily: telemedicine helping people to feel they can take control but it’s hard to measure. Teresa: Marvellous! Cindy: so watch this space. (Meeting observation, Carnford)*

### How do commissioners access information?

Commissioners acquired information through conversations, stories and documentation including online sources. Verbal exchange was particularly well suited to the fast paced, rapidly changing environment in which commissioners worked. Conversations were an important way of getting information quickly through chance encounters, formal meetings and informal gatherings. The contingent nature of these exchanges however meant that if a different combination of individuals had happened to meet, different knowledge would have been acquired and perhaps a different set of decisions would have been made.

Knowledge was also acquired through stories. A commissioning manager said that patient stories were important to maintain momentum through the lengthy, repeated decision-making cycles. Conversations and stories are different in that the first is a form of engagement with other people while the second is a way of packaging and communicating information and convincing other people. This raises an important distinction between stories as a dubious source of (anecdotal) evidence and stories as a preferred way to convey sound evidence. We found that often stories were privileged as much as other sources of information.

*And I have often thought in the past you need the story of the change. Because ideally from the time you’ve gone through health scrutiny committee, the CCG, other local groups, other stakeholder groups and especially if you get to a procurement exercise where actually you might draw services to a close and you know people may be…made redundant and there’s some heavy duty consequences for people, you need a compelling story. And often that is much more powerful than data that you want to throw at people, and so having the clinical stories is really important and the patient’s story is really, really key. (Harry, NHS commissioning manager, Deanshire)*

Although substantial knowledge acquisition occurred through informal conversation, more formal verbal exchanges such as meetings were recorded to leave a paper trail documenting discussions for accountability. Commissioners had extensive access to other documentation, much of it unsolicited and sent electronically, including national and regional directives, meeting papers, business cases, reports, patient satisfaction surveys, guidelines, pathways, and performance, activity, financial and referral data. To capture commissioners’ attention, these documents often had a summary of no more than one side of A4 with clearly bulleted action points.

### Use of academic research

Few participants mentioned accessing academic research directly, and those that did said that they searched Google and Google Scholar. Apart from the *British Medical Journal* and the *British Journal of General Practice*, no other academic journal was named. This was not surprising as commissioners were often unable to access full length articles without journal subscriptions. Participants frequently mentioned the *Health Service Journal* as a source of research evidence, although this professional journal usually showcased best practice and service innovations with only the occasional synopsis of academic research (www.hsj.co.uk).

When asked about use of academic research, participants mainly discussed the difficulties. These included:

- the challenges of finding applicable, relevant research for commissioning , given that most health related academic research was clinically focused
- “academically very robust” interventions not working in practice (Jane, NHS commissioning manager, Deanshire)
- the challenge of drawing conclusions from literature reviews with substantial variations in interventions
- lack of commissioner time and skills to do comprehensive reviews
- difficulties in interpreting the “spin” within abstracts (Mary, Public health consultant, Carnford)
- the length of time needed for locally commissioned research to produce meaningful outputs
- challenges in applying negative or inconclusive findings, as commissioners’ focus was on identifying initiatives that might work

Participants talked about academic research being implicit in the system. Clinicians and commissioners were “expected to keep on top” of their area (Alan, NHS commissioning manager, Penborough) sometimes by having their “ear to the ground” with national and local networks (Vidur, GP commissioner, Penborough). The expectation was that clinical commissioners would bring a research perspective. In an observation of one commissioning meeting, this higher level of understanding of research was visible when a GP commissioner explained ‘funnel plots’ to his colleagues.

Academic research was regarded as being implicit in national guidance, especially from professional bodies and NICE. One GP commissioner, on his own initiative, provided a regular and enthusiastically received clinical briefing that summarised academic research alongside an update on local services (e.g. referral waiting times). More generally, NICE guidance and guidelines were seen as “absolutely crucial” to commissioning decisions, although they were sometimes described as problematic - “we know we would go bust if we implemented all of them” (Patrick, GP commissioner, Norchester). Another difficulty was the absence of local services required to deliver NICE recommendations. Commissioners tended to select the “best ones” (defined as most “do-able” of NICE recommendations) (Patrick, GP commissioner, Norchester).

Public health departments were often cited as places to find academic research. For example, academic research evidence was formally presented at the unscheduled-care board meetings of one commissioning organisation which included representatives with many different viewpoints including patients. These evidence summaries were clear, simple documents consisting of a table with three columns: type of intervention, research evidence of impact, overall conclusion (i.e. effective/not effective/inconclusive). The committee welcomed to these reviews.

*Teresa comments that this presentation is very, very helpful. People are interested in more reviews of research evidence around unscheduled care, to help with things like education and self-management….. Anthony: That is the sort of input we want, and have not had in past. (Meeting notes, Carnford)*

But despite the committee’s enthusiasm, they did not know how to apply the information. For example, when faced with an evidence review on unplanned hospital admissions, the committee scanned through the interventions and briefly discussed one intervention with inconclusive evidence, which a member subsequently stated was a “good idea”. The document was then put to one side. This committee did not appear to consider the disinvestment (i.e. decommissioning) opportunities highlighted by these reviews. The public health consultant was aware of the difficulties in applying the information, especially as much of the research evidence was about ineffective interventions.

*So for instance the work [on unscheduled care] an awful lot of it was like, “Well there is no evidence that this works and there’s no evidence that that works.” And that, it’s really interesting, but then you think, well if we are trying to get people who have these really kind of strict targets, real pressure to reduce costs, and to just come in every month saying “We’ve looked at this and it doesn’t work,” then that’s quite a difficult position to be in. (Rick, public health consultant, Carnford)*

One commissioning organisation was highly enthusiastic about ‘evidence-based policy-making’ largely because of the influence of a respected academic GP with substantial influence amongst the senior directors. However instead of the insistence on research evidence facilitating policy decisions, it was sometimes a barrier. For example, if limited academic evidence was available, some commissioners from this organisation would prevaricate and avoid moving forward, even for nationally mandated directives with which local commissioners had to comply.

*…[they become] frozen because you can’t make pragmatic decisions. Because it’s like America is out there but they will not set sail until they know it’s there….So with interventions like ‘telehealth’, “they’ll say ‘well we’re not doing that.’” (Patrick GP commissioner, Norchester)*

Elsewhere commissioning organisations also struggled with their use of academic research. A GP commissioner stated that the demand for research evidence reduced innovation, because commissioners could not wait until an initiative was “piloted and proven” (Ralph, GP commissioner, Carnford). Consequently, some commissioners stated that it was permissible to fund initiatives without research evidence as long as these were couched as pilots and not much money was invested. Moreover, sometimes initiatives with negative or no evidence were funded because commissioners still needed to deliver a viable financial plan. The imperative to come up with an initiative that looked like it could save money would push it through, regardless of research that suggested the contrary (or no research at all).

*I’ve had conversations [with colleagues] about, “Well, you know, we shouldn’t be putting that down to say it will make savings because there’s no evidence that it will,” versus me saying, “But actually we’ve still got a statutory responsibility to deliver a balanced plan, and if I take those savings out they need to come from somewhere else.” (Carla, NHS commissioning manager, Norchester)*

### Local evaluations

Although use of academic research was infrequent, in our observations of commissioning meetings, commissioners repeatedly called for local evaluations of services and innovations “to say ‘yes’ and it is delivering what we expected – or it isn’t” (Clara, commissioning manager, Carnford). For example, one chair mentioned that they had eight different initiatives to address problems around delayed transfer of care, but no mechanism to find out if any were “contributing well or contributing badly to that final outcome” (Simon, CCG Chair, Norchester).

Three service evaluations in particular were mentioned by participants from three different commissioning organisations. These included a controlled cohort study of the rehabilitation project (see above and Additional file 1), a cohort study mapping the service usage of 50 patients at a local hospital and a controlled cohort study on the impact of case management on hospital usage. The latter evaluation was led by a local academic GP and was mentioned by several participants. The findings of this study were seen to have a clear impact on commissioning decisions, as the commissioning organisation decided not to expand the service, although the nature of the contract meant that it could not recoup the costs of the service.

### A vignette

To illustrate the findings made throughout the results section, we present here an example of commissioners’ information seeking and ways that this fed into the decision making process in the following box (Additional file 1).

## Discussion

### Principal findings

By juggling competing agendas, priorities, power relationships, demands and personal inclinations, commissioners built persuasive, compelling arguments to inform local commissioning decisions. They sought information to identify options, navigate a way through the system, justify decisions and convince internal and external parties to approve and/or follow the suggested course. This was the ‘art of commissioning’. To build the case, different types of ‘evidence’ from an array of sources were pragmatically selected, such as best practice guidance, clinicians’ views of services and innovations from elsewhere. Academic research was less useful, possibly because its presentation was inaccessible or because of non-committal or negative conclusions which did not contribute towards developing viable commissioning plans. Interestingly, negative academic research evidence did not appear to inform disinvestment strategies.

Information was passed through conversations and stories, which were fast, flexible and suited the rapidly changing world of commissioning. Stories were particularly forceful in conveying ideas. Local data often trumped national or research-based information and local evaluations were seen as helpful in directly answering commissioners’ questions. Our study suggests that far from being evidence-free, commissioners routinely drew on a wide range of sources to inform and support their decisions. However unlike the idealised vision of evidence-based policy-making, little of this evidence was drawn from academic research.

Previously identified barriers to research use by policymakers include the cultural and organisational gaps between researchers and policymakers, practical pressures such as timeliness and timeframes, difficulties in accessing research and lack of research skills or awareness amongst policymakers [17–20]. The two key facilitators already known to increase the use of academic research are relevant and clear information and personal contact [19, 21]. But our study reveals that even with these facilitators in place, commissioners struggled to apply research findings in their decision-making.

For example, a locally known and respected public health consultant presented clear, simple rapid evidence reviews in line with guidance on making systematic reviews more accessible for commissioners [22, 23], but with apparently limited impact. This was possibly because the review found few interventions of benefit and/ or the public health consultant did not sufficiently interpret the findings for the local context or correct the committee’s misunderstandings. In our observations of effective presentations to commissioners, a few clear, brief messages were necessary to steer the discussions, otherwise the commissioning committees did not know how to respond. In the absence of such direction, these reviews may have given commissioners more confidence in knowing that at least academic research had been consulted, but commissioners were still left with the task of developing viable financial strategies. Consequently, sometimes even initiatives with inconclusive or negative research evidence were acted on because commissioners (a) needed to do something b) did not want to dampen people’s enthusiasm c) needed to demonstrate innovation d) wanted to evaluate things within their own system and/or e) thought their implementation of the intervention was different and more likely to lead to success.

The inclusion of research evidence is generally expected to result in ‘better’ policy, although some have noted that this remains untested empirically [24]. We found that an insistence on academic research as the ultimate arbitrator of decision-making could stall, derail or “paralyse” the commissioning process. This raises an interesting question: if policymakers routinely resolve to make decisions largely based on academic research, and given that relevant academic research often does not exist or is unusable in its current format, could that actually be counter-productive?

### Strengths and weaknesses of our research

Much of the literature about ‘evidence-based policy-making’ focuses on barriers to research utilisation, usually from the viewpoint of researchers, and/ or relies largely on self-reports by policymakers in interview studies [19]. This paper reports an empirical study drawing on observations, as well as interviews and documentation, of commissioners working in their ‘real life’ settings. This gave opportunities to observe knowledge acquisition in practice and question key parties about their role, involvement, understanding and experiences. With documentary data, we were also sometimes able to track information as it moved through the system. However, we were not in the field constantly. Sometimes the information disappeared, morphed or reappeared elsewhere, without our knowledge. Although we got closer to understanding how commissioners access and use information, there are still some gaps that future empirical studies could address. For example, an ethnographic study tracking a single commissioning proposal from the early stages could be illuminating.

A further limitation in common with other ethnographic studies is that we do not know to what extent the presence of researchers changed the dynamics of the meetings observed. However, commissioning meetings often included other observers, such as external consultants or staff waiting to speak to a particular agenda item and even occasionally journalists, as some meetings were public forums. Therefore, arguably the presence of researchers had less impact than usual.

A strength of the study was the level of reflexivity and challenge built into the study design. At the beginning, we devised several questions around our assumptions and prejudices which were revisited at intervals throughout data collection and analysis. In addition to including two commissioners on the study team, who regularly came to meetings, we also convened workshops with a small group of commissioners at two time points – mid-way and at final report writing stage – to sharpen our thinking and challenge our findings. As the team developed, we became accustomed to regularly questioning each other’s interpretations, which was recorded and fed into our emerging analyses.

### Implications of findings

A tempting response to the limited use of research evidence within policy-making is to ‘correct’ commissioners by enhancing their skills in accessing and utilising research. But this ignores what we have learnt about the real nature of their work, which suggests that we may need to adapt our role as researchers. Our research suggests four key problems. First, we produce volumes of clinical research of which relatively little is immediately relevant to commissioners. Secondly, instead of relying on verbal modes of transmission, which commissioners prefer, we communicate largely through the written word, specifically peer reviewed papers which many commissioners cannot access or understand. Thirdly, we use methods designed to eliminate contextual factors (such as randomised controlled trials, surveys, cohort studies) to provide ‘generalisable’ outputs without then offering commissioners the necessary ‘local’ interpretation required. Fourthly, we make little effort to understand the context commissioners work in and why our outputs are seldom used. Instead, we continue to produce more research in the same ways and disseminate it using methods consistently shown not to work.

Dubrowa et al. make a useful distinction between the different positions of researchers and commissioners [25]. More positivist researchers tend to see “evidence and context as mutually exclusive”, therefore perceiving research evidence that is stripped of context to be of higher quality and to engender “higher quality decisions”. In contrast, the “practical-operational” orientation common amongst decision-makers views evidence as highly context dependent [25]. This need for ‘knowledge-in-practice-in-context’ has also been suggested as a key reason why clinicians find it difficult to use generalised or research-based clinical guidelines [26]. Following this line of argument, commissioners will always struggle to apply systematic reviews and randomised controlled trials, because paradoxically the very qualities that create such context-free, ‘gold standard’ research render the studies less useful to commissioners. Instead, commissioners want “killer” stories that are rich in context [27, 28]. This suggests that the ‘problem’ is less about commissioners having to change their information acquisition habits and more about the way that academics generate research. Rather than a ‘demand’ problem, we may have a ‘production’ problem [29].

This study suggests several ways for researchers to address the ‘production’ problem’. First, academic researchers need to engage with commissioners using commissioners’ preferred methods of conversations and stories, to find out what is wanted and how best to deliver it. Researchers need to stop relying mainly on written communication to get their messages across. Starting locally would make this more manageable. Secondly, researchers need to learn more about local commissioning priorities to produce more relevant, useful research. Increasingly, finding out about local priorities is easier as commissioning organisations are on-line (see e.g. https://www.bristolccg.nhs.uk/library). Thirdly, researchers need to learn to package their messages in commissioner-friendly ways. Swan et al. suggest that case studies offer context-rich information [29] that Lomas and others note are essential to inform policy-making decisions [30]. Fourthly, researchers can build relationships with intermediaries with their local public health departments whose staff understand the value of research and have wide commissioner networks. In developing such relationships, academics could also proactively disseminate case studies of ‘best practice’ to national bodies such as the Royal Colleges and NHS Improving Quality. Finally, researchers have a wealth of useful methodological knowledge that commissioners would find valuable in their local evaluations or needs assessments. As Oliver et al. suggest, “If universities were to provide assistance for local policymakers in the analysis of existing data, a relationship of mutual benefit could start to develop” [19].^1^

## Conclusion

In this study of commissioners’ information seeking behaviour which aimed to inform the ways that researchers’ communicate their knowledge, we found that above all commissioners were pragmatic. They accessed and incorporated whatever information helped to build a cohesive, compelling case to inform decision-making. In considering use of research evidence, the problem was not that commissioners did not want to use academic research (in fact there was enthusiasm). Rather that commissioners tended to view academic research as not often making a useful contribution and so they sought more helpful information elsewhere. If researchers are to contribute to commissioners’ decision-making, then we need to produce more useful information and develop relationships of mutual benefit through what van de Ven calls “engaged scholarship” [31]. This is more likely to happen through utilising commissioners’ preferred communication mode of conversations and stories and contributing academics’ skills and time to designing and conducting co-produced local evaluations steeped in the actual context of commissioning.

## Additional file

Additional file 1:
**Vignette of commissioners’ information seeking behaviour.** (DOCX 13 kb)

## Footnotes

1 This is starting to happen in southwest England. A Knowledge Mobilisation team, led by the lead author and made up of commissioners and ‘researchers in residence’ seconded into each other’s organisations, is currently developing relationships through conducting jointly co-produced service evaluations. This is funded by ‘research capability funding’ and sponsored by Avon Primary Care Research Collaborative, a local brokering organisation that sits within local clinical commissioning groups and supports researchers and commissioners.

## References

[1] Ham C, Hunter D, Robinson R. Evidence based policy making. BMJ 1995*.* 1995, 310(71)

[2] Oxman A, Lavis J, Lewin S, Fretheim A. SUPPORT Tools for evidence-informed health Policymaking (STP) 1: What is evidence-informed policymaking? Health Research Policy and Systems*.* 2009, 7(Suppl 1). 10.1186/1478-4505-7-S1-I1 PMC3271819 20018098

[3] Cookson R (2005). Evidence based policy making in healthcare: what it is and what it means. J Health Serv Res Policy.

[4] Klein R (2000). From evidence based medicine to evidence based policy?. J Health Serv Res Policy.

[5] Department of Health (2010). Liberating the NHS.

[6] Nutley S, Walter I, Davies H (2007). Using Evidence: how research can inform public services.

[7] Checkland K, Harrison S, Snow S, Coleman A, McDermott I (2013). Understanding the work done by NHS commissioning managers: an exploration of the microprocesses underlying day-to-day sensemaking in UK primary care organisations. J Health Organ Manag.

[8] Shaw SE, Smith JA, Porter A, Rosen R, Mays N. The work of commissioning: a multisite case study of healthcare commissioning in England’s NHS. BMJ Open. 2013, 3(9). 10.1136/bmjopen-2013-003341 PMC3773628 24014483

[9] Gkeredakis E, Swan J, Powell J, Nicolini D, Scarborough H, Roginski C, Taylor-Phillips S, Clarke A (2011). Mind the gap: understanding utilisation of evidence and policy in health care management practice. J Health Organ Manag.

[10] Gabbay J, le May A, Pope C, Robert G (2011). Organisational innovation in health services: lessons from the NHS Treatment Centres.

[11] Checkland K, Snow S, McDermott I, Harrison S, Coleman A, et al. ‘Animateurs’ and animation: what makes a good commissioning manager? Journal of Health Services Research & Policy. 2012;17:11-7. 10.1258/jhsrp.2011.011010 21967825

[12] Wye L, Brangan E, Cameron A, Gabbay J, Klein J, Pope C. Knowledge exchange in healthcare commissioning: case studies of the use of commercial, not-for-profit and public sector agencies, 2011–14. Health Service Delivery Research 2015, 3(19). 25950073

[13] Wye L, Brangan E, Cameron A, Gabbay J, Klein J, Anthwal R, Pope C. What do external consultants from private and not-for-profit companies offer healthcare commissioners? A qualitative study of knowledge exchange. BMJ Open*.* 2015, 5(e006558). 10.1136/bmjopen-2014-006558 PMC4342588 25716174

[14] Yin R (2002). Case Study Research.

[15] Patton M (2002). Qualitative Research and Evaluation Methods.

[16] Clarke A, Taylor-Phillips S, Swan J, Gkeredakis E, Mills P, Powell J, et al. Evidence-based commissioning in the English NHS: who uses which sources of evidence? A survey 2010/2011. BMJ Open 2013, 3(5) 10.1136/bmjopen-2013-002714 PMC3657651 23793669

[17] Orton L, Lloyd-Williams F, Taylor-Robinson D, O’Flaherty M, Capewell S. The use of research evidence in public health decision making processes: systematic review. PLoS One*.* 2011, 6(7). 10.1371/journal.pone.0021704 PMC3144216 21818262

[18] Innvaer S, Vist G, Trommald M, Oxman A (2002). Health policy-makers’ perceptions of their use of evidence: a systematic review. J Health Serv Res Policy.

[19] Oliver K, Lorenc T, Innvaer S. New directions in evidence-based policy research: a critical analysis of the literature. Health Research Policy and Systems*.* 2014, 12(34). 10.1186/1478-4505-12-34 PMC4107868 25023520

[20] Mitton C, Patten S (2004). Evidence based priority setting: what do the decision makers think?. J Health Serv Res Policy.

[21] Dobbins M, Rosenbaum P, Plews N, Law M, Fysh A (2007). Information transfer: what do decision makers want and need from researchers?. Implement Sci.

[22] Lavis J, Davies H, Oxman A, Denis JL, Golden-Biddle K, Ferlie E (2005). Towards systematic reviews that inform health case management and policy making. J Health Serv Res Policy.

[23] Chambers D, Wilson PM, Thompson CA, Hanbury A, Farley K, Light K (2011). Maximizing the impact of systematic reviews in health care decision making: a systematic scoping review of knowledge-translation resources. Milbank Q.

[24] Cameron A, Salisbury C, Lart R, Stewart K, Peckham S, Calnan M, Purdy S, Thorp H (2011). Policy makers’ perceptions on the use of evidence from evaluations. Evidence & Policy.

[25] Dubrowa M, Goelb V, Upshurc R (2004). Evidence-based health policy: context and utilisation. Soc Sci Med.

[26] Gabbay J, le May A (2011). Practice-Based Evidence for Healthcare.

[27] Macintyre S (2012). Evidence in the development of health policy. Public Health.

[28] Stevens A (2011). Telling policy stories: an ethnographic study of the Use of evidence in policy-making in the UK. J Soc Policy.

[29] Swan J, Clarke A, Nicolini D, Powell J, Scarbrough H, Roginski C, Gkeredakis E, Mills P, Taylor-Phillips S (2012). Evidence in management decisions (EMD) - advancing knowledge utilisation in healthcare management.

[30] Lomas J (2005). Using research to inform healthcare managers’ and policy makers’ questions: from summative to interpretive synthesis. Healthcare Policy.

[31] Van de Ven A (2007). Engaged Scholarship: A Guide for Organizational and Social Research.

