# Supplementary material for: Evidence based policy making and the ‘art’ of commissioning – how English healthcare commissioners access and use information and academic research in ‘real life’ decision-making: an empirical qualitative study
Source: BMC Health Serv Res. 2015 Sep 29;15:430. doi: 10.1186/s12913-015-1091-x (PMC4587739; doi:10.1186/s12913-015-1091-x)
Supplement: Additional file 1: — Vignette of commissioners’ information seeking behaviour. (DOCX 13 kb) [file 12913_2015_1091_MOESM1_ESM.docx]

| **Additional file 1 Vignette of commissioners’ information seeking behaviour**  Both the local healthcare commissioners and local authority had identified rehabilitation as a priority, in response to national government initiatives (e.g. Transforming Community Services). As part of the ‘check phase’ at the beginning, the project team ‘horizon scanned’ to find out how organisations elsewhere were approaching the task of re-designing rehabilitation services, without much success.  *There didn’t seem to be any sort of traditional clinical research. There was sort of service evaluation type research being carried out across the UK as well as elsewhere…our public health colleagues undertook a literature review and evaluation of what else we knew, what else was going on. And it’s incredibly difficult to draw any conclusions, because no two services are the same, and certainly weren’t using the same measures or outcomes and so on, to be able to draw conclusions from.… they probably told us more about what we didn’t want to do than what we wanted to emulate, which is why we wanted so much to take this in a different direction. (Jane, NHS commissioning manager)*  One of the local authority managers instigating the rehabilitation project thought that a change management method used by a consultancy firm working on another programme was applicable. The local authority had access to funding and the consultancy firm was hired to help.  *We used their method of system redesign, which was about working out what your purpose was…. it was really a good method to use actually because every time we got a bit lost we said right we’ll go back and identify what the purpose was decided by the patients, and so it was completely redesigned from a blank sheet of paper… it was kind of a really good example of how patient centred commissioning should be done I think. (Lynn, NHS clinical commissioner)*  A multi-disciplinary team was formed whose information-gathering activities included shadowing care professionals and joint visits to service users’ homes to talk to them about their experiences. The consultancy firm carried out detailed mapping of a small number of one-year patient journeys, including costs and outcomes. A commissioning manager described this as a turning point for engaging clinicians and providers in the project:  *And it was really shocking for providers to see that whole picture from the individual’s perspective. And one gentleman had been assessed over thirty-five times … Somebody else had had about eighty treatments of one nature or another… (Jane, NHS commissioning manager*)  A set of very powerful patient stories emerged, which were used during the commissioning process to illustrate what was wrong with the old system and motivate people to take bold steps to change it. A radically different, service-user-centred model was designed and piloted. More information was then needed to decide what to do next:  *We have done our best to evaluate the pilot, and we worked with public health and it was really, really difficult because you know in health we’re not very good at evaluating things because it’s so nebulous. And how can you tell when you have lots of initiatives, we’re very good at doing initiatives, what is it that’s made the difference? And we did try as hard as we could, and there was some statistically significant evidence that we began to build on, I mean it wasn’t huge, again there’s all sorts of flaws and it was a weak study in lots of ways, but it was something. (Abbie, NHS commissioning manager)*  Feedback from staff and service users was also gathered, and “certainly on both those counts it’s a very positive picture” (John, local authority manager). They decided to expand the area covered by the pilot, and subsequently to roll out the new service out across the whole local area.  *I think the key thing was an act of faith at the most senior level that this was the right thing to do. And we hoped that it would deliver savings, but that what we were doing wasn’t going to work in the long-term anyway, it was an outmoded model, and we needed to be doing something different. So there was a very strong – you know, I have to say, when I got here and I saw what they were trying to do and the way they were going about it, my mouth really dropped open. It was one of the bravest decisions I’ve seen, I think, in terms of an approach. And there still are major concerns about its financial viability going forward. Because it isn’t yet delivering the savings that we might hope, and that could be because the model doesn’t work or it could be because it does work but it’s more expensive. Or it could be, and this is what we’re holding onto, that it’s because it’s only – it’s still only – it’s been going in one part of the county for 18 months, another part for six months, and actually the bulk of our cost is driven by people who haven’t been through the service. (John, local authority manager)* |
| --- |
